# Phase I/IIa Study of an Intravitreal Optogenetic Therapy (AGN-151597) in Patients with Advanced Retinitis Pigmentosa

**DOI:** 10.1016/j.xops.2026.101323

**Published:** 2026-07-13

**Authors:** Bill Bunnell, Francisco J. López, Daisy Yuan, Michael R. Robinson, Jacque L. Duncan, David G. Birch

**Affiliations:** 1 AbbVie Inc., North Chicago, Illinois; 2 University of California, San Francisco, California; 3 Retina Foundation of the Southwest, Dallas, Texas

**Keywords:** AGN-151597, Retinitis pigmentosa, RST-001, Optogenetics

## Abstract

**Objective:**

Retinitis pigmentosa is an incurable, inherited retinal disease that causes vision loss and blindness. We evaluated the safety and preliminary efficacy of AGN-151597, a mutation-independent optogenetic therapy encoding channelrhodopsin-2, in patients with advanced retinitis pigmentosa.

**Design:**

First-in-human, phase I/IIa, open-label, dose–escalation study (1 study eye/participant).

**Participants:**

Participants had advanced retinitis pigmentosa with severely impaired visual function.

**Methods:**

Participants received an intravitreal injection of low-dose (4.3 × 10^10^ vg/eye; n = 3), mid-dose (1.4 × 10^11^ vg/eye; n = 4), or high-dose (4.3 × 10^11^vg/eye; n = 7) AGN-151597 in the study eye. The study duration was 24 months followed by a 3-year safety extension.

**Main Outcome Measures:**

The primary safety endpoint was safety at 6 months as assessed by intraocular pressure and changes in visual acuity, full-field sensitivity, ambulation, and visual anatomical parameters. Efficacy measures included changes in visual acuity, full-field stimulus threshold (FST), ambulation, object detection/discrimination, visual evoked potentials, electroretinography, and visual function-related quality of life (questionnaire).

**Results:**

Fourteen patients (median age 62 years) were enrolled. AGN-151597 treatment resulted in no clinically significant changes in ocular safety assessments. Most treatment–emergent adverse events (TEAEs) were ocular; none were severe. The most common treatment-related TEAE was transient increased intraocular pressure (n = 3). No clinically significant efficacy was observed. Full-field stimulus threshold measurements in both eyes showed good agreement between the screening and baseline visits, with coefficients of repeatability of 6.2 for blue light, 2.7 for red light, and 5.3 for white light. Overall mean FST in study eyes at baseline was –13.5 dB for blue light, –1.0 dB for red light, and –5.2 dB for white light. Participants’ mean FST changed similarly in both eyes over 24 months for each light stimulus.

**Conclusions:**

There was no evidence of AGN-151597 efficacy in slowing vision decline in patients with advanced retinitis pigmentosa. However, the absence of safety concerns over 5 years after intravitreal administration of an adeno-associated virus type 2 vector–delivered genetic medicine is encouraging and supports further gene therapy endeavors to enhance visual function in this devastating retinal disease. Full-field stimulus threshold is a reliable test in patients with poor vision.

**Financial Disclosure(s):**

Proprietary or commercial disclosure may be found in the Footnotes and Disclosures at the end of this article.

Retinitis pigmentosa is an inherited neurodegenerative disease and one of the most common and most severe causes of retinal degeneration.^1^ This deeply disabling disease affects up to 1 in 4000 people worldwide, and so far, cannot be cured.^2^ A progressive loss of photoreceptors affects rods first, then cones, followed by atrophy of the retinal pigment epithelium.^2^^,^^3^ Initially, retinitis pigmentosa manifests as night blindness, followed inexorably by continuous vision loss. As more rod cells die, the peripheral visual field progressively contracts and tunnel vision develops. Cone cells also begin to die, and eventually even the central visual field is lost, resulting in total blindness.^2^, ^3^, ^4^ Cystoid macular edema develops in approximately half of patients and worsens their remaining visual acuity.^2^

Retinitis pigmentosa is a family of single-gene disorders, often diagnosed in adolescents and young adults.^3^^,^^4^ Over 100 pathogenic genes affecting various biological pathways have been identified, mostly specific to rod cells that are essential for vision in low-light conditions.^3^ The inheritance of retinitis pigmentosa generally falls into 3 classes: autosomal-dominant, autosomal-recessive, or X-linked. The latter usually causes the most severe phenotype.^3^

The best-studied mutations causing retinitis pigmentosa are those affecting the *RHO* gene, which occur in 25%–30% of autosomal-dominant cases. *RHO* encodes rhodopsin, a G-protein–coupled receptor located in the outer segment of rod cells. When stimulated by light, it activates the phototransduction cascade. The most common mutation in this gene leads to protein misfolding. Misfolded proteins are retained in the rod cells’ endoplasmic reticulum, ultimately leading to apoptosis.^2^

Retinitis pigmentosa is incurable, but early diagnosis and intervention can slow its progression.^3^ However, no standard treatment exists, and most patients are limited to supportive care, including use of vitamin supplements and visual aids, and protection from sunlight.^5^ Although there are no available treatments to restore patients’ vision, promising medical advances—particularly with gene therapies that may be able to restore rod cells’ healthy phenotype, and optogenetic therapies that aim to restore light sensitivity to degraded retinas—mean that the possibility of halting vision loss may be within reach.^3^^,^^6^

Currently, Luxturna (voretigene neparvovec) is the only approved gene therapy for retinitis pigmentosa, yet it is authorized only for a small subpopulation of patients who have retinal degeneration associated with pathogenic variants in the *RPE65* gene, which account for <1% of patients with rod–cone degeneration. Over 100 drugs are in development for retinitis pigmentosa globally, of which half are advanced therapies such as gene, stem cell, and optogenetic therapies. Several of these are in late-stage development, including GS-030, vMCO-1, and human stem cell therapies jCell and ReN-003.^5^

AGN-151597 (formerly RST-001) was developed as a potential mutation-independent optogenetic therapy that could enable retinal cells to become light-sensitive and improve visual function in patients with retinitis pigmentosa. It encodes the channelrhodopsin-2 protein in a nonreplicating recombinant adeno-associated virus type 2 (AAV2) vector for delivery. Studies in animals with severe retinal degeneration have shown that intravitreal administration of AGN-151597 transduces retinal ganglion cells to produce channelrhodopsin-2, which depolarizes the ganglion cells in response to light, resulting in a visual signal being delivered to the brain (AbbVie, data on file). Nonclinical pharmacology and toxicology studies evaluating RST-001 in mice, rats, and dogs have shown an acceptable safety profile with no evidence of dose-limiting toxicities or a significant immune response (AbbVie, data on file).

Here, we report a first-in-human, phase I/IIa study assessing the safety, tolerability, and preliminary efficacy of intravitreal AGN-151597 in patients with advanced retinitis pigmentosa. While this study was in progress, Allergan, the study sponsor, became a subsidiary of AbbVie, and the development of AGN-151597 was discontinued for business reasons. Screening and enrollment for this study were therefore stopped, but patients already enrolled continued in the study per protocol and were followed for 5 years.

## Methods

### Design

This was a phase I/IIa, open-label, dose–escalation study. The study complied with the principles of the Declaration of Helsinki, the International Council for Harmonisation guidance, Good Clinical Practice standards, and all applicable laws and regulations. The protocol was approved by an institutional review board at each study site (WCG institutional review board, DUHS institutional review board, and University of California, San Francisco Human Research Protection Program), and all participants provided written informed consent. This study is registered on ClinicalTrials.gov with the identifier NCT02556736.^7^

### Participants

Study participants were adults with retinitis pigmentosa and severely impaired visual function. Inclusion criteria included a clinical diagnosis of advanced retinitis pigmentosa, documented retinal electrophysiological evidence of rod–cone photoreceptor degeneration, and evidence of a <10 μV maximal b-wave electroretinography (ERG) response. Retinal ganglion cells and/or the retinal nerve fiber layer were required to be present on spectral-domain OCT (SD-OCT).

Participants in phase I were required to have visual acuity in the study eye of no-better-than hand motion. If the Data Safety Monitoring Committee identified a treatment benefit in phase I, the same criteria were to apply in phase IIa. If no treatment benefit was identified, visual acuity eligibility criteria were planned to match those of phase I for half of the enrolled patients, and for the other half, visual acuity in the study eye ranging from no-worse-than count fingers to 20/200 was required.

Patients were excluded if they had other pre-existing eye conditions; had undergone prior gene or stem cell therapy, cataract surgery, or vitrectomy in the study eye; had aphakia in the study eye; had taken antiplatelet or anticoagulant medications within 28 days of treatment administration; or were taking immunosuppressant medication. Full eligibility criteria are listed in Appendix S1 (available at https://www.ophthalmologyscience.org).

### Intervention

In phase I, 3 groups of 3 patients were planned to be enrolled sequentially: group A (low dose; 4.3 × 10^10^ vg/eye); group B (mid dose; 1.4 × 10^11^ vg/eye); and group C (high dose; 4.3 × 10^11^ vg/eye). The Data Safety Monitoring Committee reviewed the emerging safety and tolerability data for at least a month after the first patient was dosed in each group, then 1 month after the remaining patients were dosed in each group, before doses were escalated. In phase IIa, approximately 12 participants were planned to be enrolled at the maximum tolerated dose identified in phase I.

AGN-151597 was administered in the study eye (1 study eye per patient) by a single 100-μL intravitreal injection. Patients were permitted to take prescribed and over-the-counter medications, as needed, unless prohibited by the protocol (Appendix S1).

Twelve core study visits were planned over 24 months, including screening and baseline visits, a day 0 (treatment) visit, and follow-up visits on days 1 and 7 and months 1, 3, 6, 9, 12, 18, and 24. After completing the core study visits, each patient was asked to enroll in a long-term follow-up for an additional 3 years to monitor the long-term safety of AGN-151597. These visits included yearly office visits for 3 years at months 36, 48, and 60.

### Objectives and Endpoints

The primary objective was to evaluate the safety of a single intravitreal injection of AGN-151597. The secondary objectives were to establish the maximum tolerated dose of AGN-151597 and to evaluate its preliminary efficacy.

The primary endpoint was safety at 6 months, as assessed by change in visual function (visual acuity and full-field sensitivity) in the study eye, change in ambulation, intraocular pressure (IOP) in the study eye, and change in anatomical parameters in the study eye on fundus photography and SD-OCT. Secondary endpoints were changes in visual acuity, full-field stimulus threshold (FST), and ambulation at 3, 12, and 24 months; object detection and discrimination, visual evoked potentials, and quality of life at 3, 6, and 24 months; ERG at 6 and 24 months; retinal appearance on fundus photography at 3 and 24 months; and SD-OCT at 3, 12, and 24 months.

Safety assessments included adverse events and other measures of ocular and systemic safety. Ocular safety measures included visual acuity, ocular inflammation, IOP, full-dilated slit-lamp biomicroscopy, color fundus photography, and autofluorescence, ERG, visual evoked potentials, FST, and SD-OCT of the macula. Systemic safety measures included physical and laboratory examination findings and vital signs. Adverse events were coded using the Medical Dictionary for Regulatory Affairs nomenclature, version 21.0, or newer. Seriousness, severity, and relationship to study drug or procedures were determined by the investigator. Severity was graded as mild (awareness of sign or symptom but easily tolerated), moderate (discomfort enough to cause interference with usual activity), or severe (incapacitating with inability to work or do usual activity).

Full-field sensitivity also was measured as part of the preliminary assessment of efficacy. Full-field stimulus threshold testing is a technique that involves presenting the patient with a series of blue, red, or white light flashes of varying intensities, with the value of 0 dB set as 0.1 cd s/m^2^ (–1.0 log cd.s/m^2^). The patient responds when flashes of varying intensity are seen, and the lower limit of light perception can be determined.^8^ Full-field stimulus threshold testing was done using the Espion^3^ ColorDome LED full-field stimulator (Diagnosys LLC) with blue, red, and white light stimuli.^9^ The maximum luminance for the white flash stimulus was 2000 cd.s/m^2^. The repeatability of FST testing was evaluated using measurements at screening (test) and baseline (retest, separated from screening by 33–85 days).

The 3 light stimuli served distinct roles. Blue light was the primary measure, because ChR2 is most sensitive to this color. Red light served as a within-patient negative control. ChR2 is expected to be least sensitive to red light so an improvement under blue but not red light would have provided strong evidence of a ChR2-specific treatment effect, while parallel changes across both colors would have suggested nonspecific influences. White light was included as it contains the full visible spectrum and is the test used in clinical practice. Together, the 3 stimuli allowed assessment of both the specificity and clinical relevance of any treatment response.

Other efficacy assessments included visual acuity, ambulation, object detection and discrimination, visual evoked potentials, ERG, and vision-related quality of life on the 25-item National Eye Institute Visual Function Questionnaire.^10^ Details of the measurements of each of the safety and efficacy parameters are provided in Appendix S2 (available at https://www.ophthalmologyscience.org).

### Statistical Analysis

The sample size for this study was chosen empirically; no formal computations for power were made. A sample size of approximately 3 patients in each dose group is typical for phase I dose–escalation studies.

Statistical analyses used SAS version 9.4 (SAS Institute Inc). All analyses used observed data in the safety population of all patients who signed the informed consent, were assigned a participant ID number, and received an intravitreal injection of AGN-151597.

Changes in FST were evaluated across all participants regardless of dose group and study phase because of the small sample sizes. Analysis of the relationship between test (screening) and retest (baseline) FST measurements in the study eye used Spearman rank correlation. The agreement in FST results between test and retest was further evaluated with Bland–Altman analysis.

Comparisons of FST results between treated (study) and untreated (fellow) eyes were conducted ad hoc. Random coefficient modeling was used to evaluate the rates of change in FST sensitivity in treated and untreated eyes. Specifically, a linear mixed-effects model was fitted, including random intercepts and random slopes for time nested within subject/eye/color. Fixed effects included baseline, time, eye, color, and all their interactions.

## Results

Safety was evaluated through 5 years of follow-up after a single intravitreal injection of AGN-151597. The evaluation of preliminary efficacy focused on FST and the repeatability of the FST measurements in this population with very poor vision.

### Disposition and Demographics

Fourteen participants were enrolled. Ten were enrolled sequentially in phase I to receive the low (n = 3), mid (n = 4), or high (n = 3) dose, and 4 were enrolled in phase IIa to receive the maximum tolerated dose, which had been determined to be the high dose. Three participants discontinued the core study (2 withdrew consent and 1 experienced a fatal adverse event of cardiac arrhythmia considered unrelated to study treatment). Nine participants entered the long-term follow-up, of whom 7 completed it (2 were lost to follow-up).

Most participants were male (n = 10; 71.4%) and White (n = 11; 78.6%). The median age was 62 years (range, 26–84; Table 1). The overall mean FST at baseline in study eyes was –13.5 dB for blue light, –1.0 dB for red light, and –5.2 dB for white light.

**Table 1:** Baseline Demographics (Safety Population)

Parameter	Phase I Low Dose (N = 3)	Phase I Mid Dose (N = 4)	Phase I High Dose (N = 3)	Phase IIa High Dose (N = 4)	Overall (N = 14)
Median age (range), years	64 (50–84)	58.5 (54–64)	62 (58–74)	49.5 (26–75)	62 (26–84)
Age, years					
<45	0	0	0	2 (50.0)	2 (14.3)
45–65	2 (66.7)	4 (100.0)	2 (66.7)	0	8 (57.1)
>65	1 (33.3)	0	1 (33.3)	2 (50.0)	4 (28.6)
Sex, n (%)					
Male	2 (66.7)	2 (50.0)	3 (100.0)	3 (75.0)	10 (71.4)
Female	1 (33.3)	2 (50.0)	0	1 (25.0)	4 (28.6)
Race/ethnicity, n (%)					
White	1 (33.3)	4 (100.0)	3 (100.0)	3 (75.0)	11 (78.6)
Black or African American	1 (33.3)	0	0	1 (25.0)	2 (14.3)
Asian	1 (33.3)	0	0	0	1 (7.1)
Hispanic or Latino	0	0	0	0	0

### Safety

A single intravitreal injection of AGN-151597 resulted in no clinically significant changes in ocular safety assessments over the 5-year period (core study and extended follow-up), including change from baseline in full-field sensitivity, ambulation, object detection, visual evoked potentials, ERG, IOP, SD-OCT, and 25-item National Eye Institute Visual Function Questionnaire composite score.

Color fundus photography and autofluorescence evaluations showed no evidence of retinal hemorrhage or detachment in any dose group. However, most participants had evidence of retinal pigment epithelium disturbance at baseline and months 3, 6, and 24.

Most participants experienced ≥1 treatment–emergent adverse event (TEAE; Table 2). Most TEAEs were ocular and were reported in the study eye. No serious or severe ocular TEAEs were reported. Two participants had nonocular serious TEAEs that were determined to be unrelated to treatment (a patient in the low-dose group had serious TEAEs of intestinal obstruction, postprocedural hemorrhage [secondary to biopsy], musculoskeletal chest pain, and cerebrospinal fluid leakage deemed unrelated to treatment, and a patient in the phase IIa group had a serious TEAE of arrythmia deemed unrelated to the study treatment that led to death).

**Table 2:** Overview of Adverse Events (Safety Population)

Adverse Event, n (%)	Phase I Low Dose (N = 3)	Phase I Mid Dose (N = 4)	Phase I High Dose (N = 3)	Phase IIa High Dose (N = 4)
Any TEAE	2 (66.7)	3 (75.0)	2 (66.7)	3 (75.0)
Ocular TEAE	1 (33.3)	1 (25.0)	2 (66.7)	3 (75.0)
Study eye ocular TEAE	0	1 (25.0)	2 (66.7)	3 (75.0)
Nonocular TEAE	1 (33.3)	2 (50.0)	0	0
Treatment-related TEAE	0	1 (25.0)	1 (33.3)	3 (75.0)
Ocular	0	1 (25.0)	1 (33.3)	3 (75.0)
Nonocular	0	0	0	0
Mild	0	1 (25.0)	1 (33.3)	1 (25.0)
Moderate	0	0	0	2 (50.0)
Severe	0	0	0	0
Serious TEAE	1 (33.3)	0	0	1 (25.0)
Death	0	0	0	1 (25.0)∗

TEAE = treatment–emergent adverse event.

∗ This patient experienced a sudden cardiac arrhythmia 491 days after dosing that led to death on the same day.

All treatment-related TEAEs that were reported were ocular (Table 3) and were mild or moderate in severity, and all except increased IOP were reported in a single participant. Treatment-related increased IOP was reported in 3 participants (each in a high-dose group) and was transient, having resolved after 1 day. Inflammation-related treatment-related TEAEs were also reported in a total of 3 participants in the high-dose groups (1 participant with anterior chamber cell that resolved without treatment within 6 days, 1 participant with iritis and vitritis treated with topical and intravitreal corticosteroid that resolved within 2 months, and 1 participant with vitreal cells that resolved without treatment within 1 week and optic neuritis treated with intravitreal triamcinolone acetonide that resolved with sequelae after 8 months).

**Table 3:** Treatment-Related TEAEs

TEAE, n (%)	Phase I Low dose (N = 3)	Phase I Mid dose (N = 4)	Phase I High dose (N = 3)	Phase IIa High dose (N = 4)
Intraocular pressure increased	0	0	1 (33.3)	2 (50.0)
Anterior chamber cell	0	0	1 (33.3)	0
Conjunctival hemorrhage	0	0	1 (33.3)	0
Eye irritation	0	0	0	1 (25.0)
Eye pain	0	1 (25.0)	0	0
Iritis	0	0	0	1 (25.0)
Optic neuritis	0	0	0	1 (25.0)
Vitreal cells	0	0	0	1 (25.0)
Vitreous detachment	0	0	0	1 (25.0)
Vitritis	0	0	0	1 (25.0)

TEAE = treatment–emergent adverse event.

The number of participants evaluated in each dose group was too low to identify dose-dependent safety signals from any safety assessments. Five of the 14 participants developed neutralizing antibodies to the AAV2 virus capsid.

### Full-Field Sensitivity

**Figure 1:**
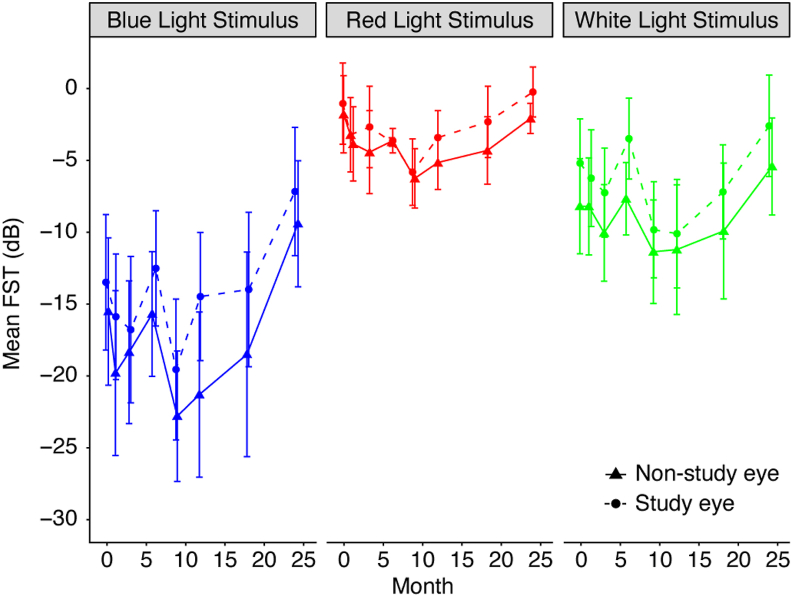
Mean FST (dB) over 24 months for each light stimulus. The number of patients at each timepoint ranged from 5 to 12. Error bars represent the standard error of the mean. FST = full-field sensitivity threshold.

**Figure 2:**
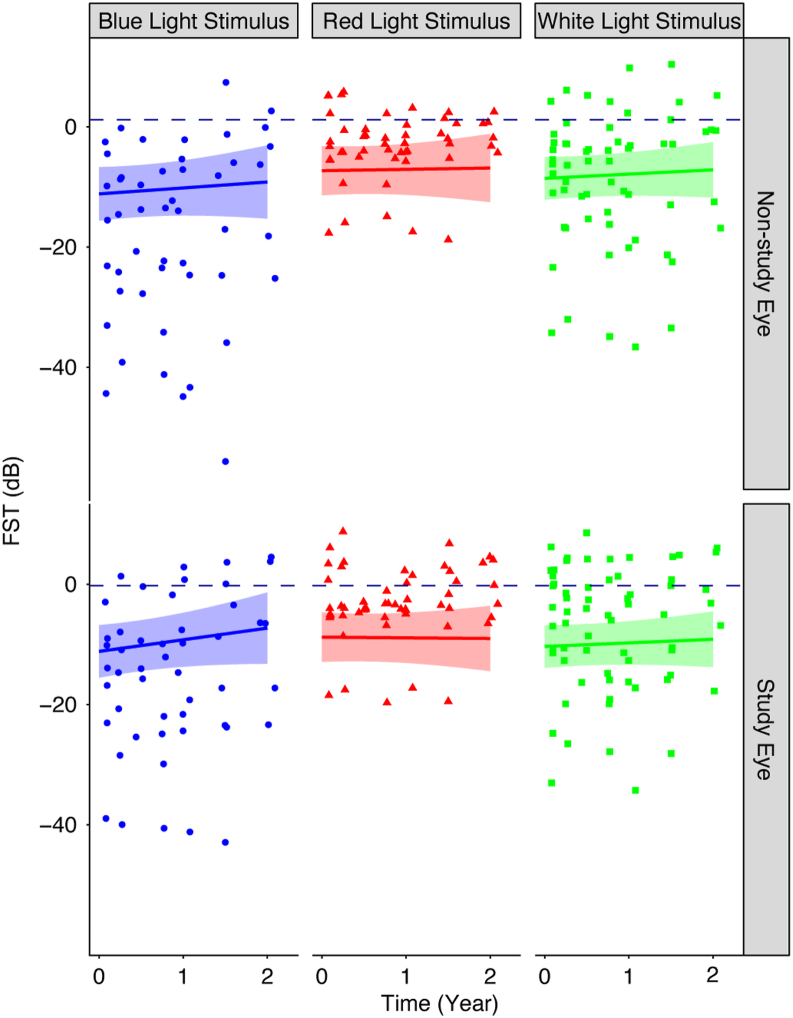
Full-field sensitivity threshold (dB) over 24 months for each light stimulus. Symbols show threshold values for individual patients, with the number of patients at each timepoint ranging from 5 to 12. Lines show the modeled trend line with a shaded two-sided 95% confidence interval band. FST = full-field sensitivity threshold.

No clinically significant effect of AGN-151597 on full-field sensitivity was evident. After AGN-151597 administration, both treated and untreated eyes showed similar full-field sensitivity changes over 24 months (Fig 1). Both treated and untreated eyes were most sensitive to the blue light stimulus and were least sensitive to (and showed the least variability in thresholds for) the red light stimulus (Fig 1). With each light stimulus, changes from baseline in FST over 24 months were minimal. The rates of change in FST values were similar for treated and untreated eyes (Fig 2, Table 4). As the 95% confidence intervals for the rates of change include zero, none of the rates of FST change during the assessment period were significant, suggesting that FST remained stable.

**Table 4:** Rate of Change in FST

Stimulus	Rate of Change in FST (95% CI), dB/Year	
	Study Eye (N = 14)	Nonstudy Eye (N = 14)
Blue light	1.93 (–0.62 to 4.47)	0.99 (–1.62 to 3.61)
Red light	–0.09 (–2.39 to 2.22)	0.24 (–2.22 to 2.70)
White light	0.61 (–1.31 to 2.53)	0.73 (–1.19 to 2.64)

CI = confidence interval; FST = full-field sensitivity threshold.

**Figure 3:**
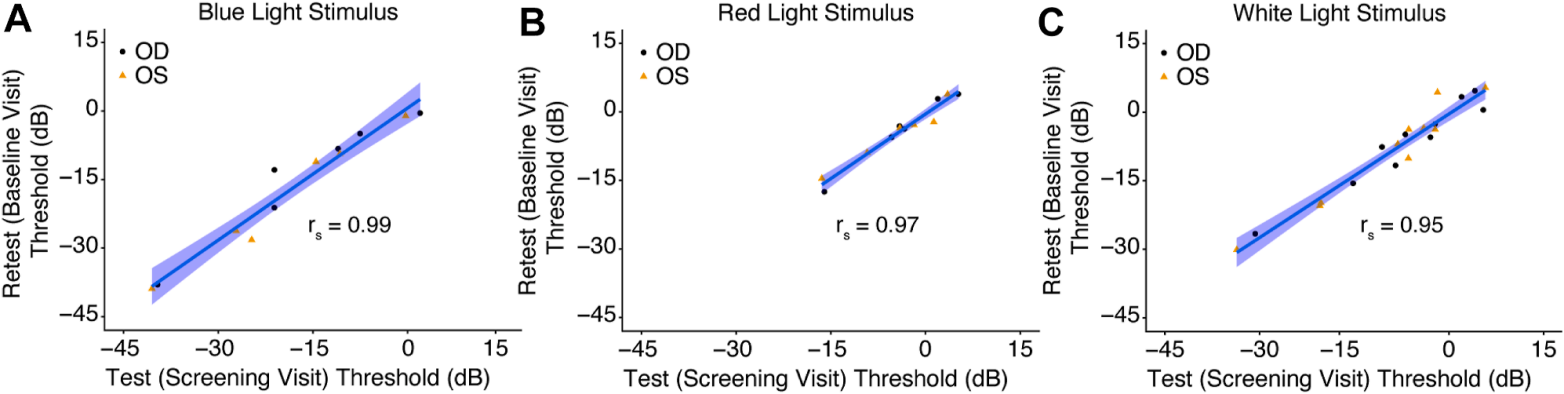
Test–retest correlation for full-field stimulus thresholds to (**A**) blue, (**B**) red, and (**C**) white light stimuli. OD = right eye; OS = left eye; R_s_ = Spearman rank correlation coefficient.

Full-field stimulus threshold measurements in both eyes showed good agreement between the screening and baseline visits, with coefficients of repeatability of 6.2 dB for blue light, 2.7 dB for red light, and 5.3 dB for white light. Results at the screening (test) and baseline (retest) visits were strongly correlated (Fig 3). Mean sensitivity thresholds at test/retest were –15.6 dB/–16.0 dB, –4.9 dB/–1.5 dB, and –9.7 dB/–7.7 dB, respectively, for blue, red, and white light.

**Figure 4:**
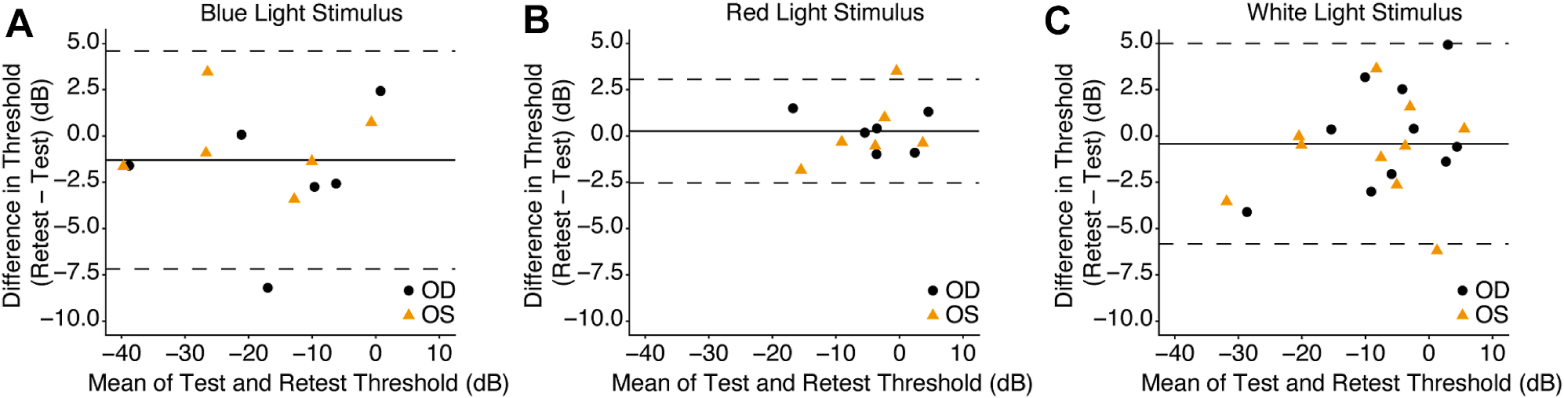
Bland–Altman plots of the agreement between test (baseline) and retest (screening) full-field stimulus thresholds for (**A**) blue, (**B**) red, and (**C**) white light stimuli. The solid line shows the average difference between test and retest measurements; the dashed lines show the 95% CI of the difference. CI = confidence interval; OD = right eye; OS = left eye.

Figure 4 shows Bland–Altman plots of the difference in threshold sensitivity in the study eye between the screening and baseline measurements (retest minus test) against the average threshold sensitivity across screening and baseline. The mean difference between screening and baseline thresholds was approximately –1.5 dB for blue light and approximately 0 dB for red and white light.

### Other Efficacy Parameters

No clinically significant efficacy of AGN-151597 was observed up to month 24 in visual acuity, ambulation, object detection and discrimination, visual electrophysiological measures, or vision-related quality of life. Data on ambulation were inconclusive and are shown by dose group in Appendix S3 (available at https://www.ophthalmologyscience.org).

## Discussion

A single intravitreal injection of AGN-151597 resulted in no clinically significant changes in ocular safety assessments over a 5-year period, including visual acuity, full-field sensitivity, ambulation, object detection and discrimination, visual evoked potentials, ERG, IOP, and quality-of-life scores. There were also no indications of clinically significant efficacy of AGN-151597 in visual acuity, full-field sensitivity, ambulation, object detection and discrimination, and visual electrophysiological measures up to month 24. Although the untreated fellow eye was not used as a formal control for study purposes, evaluation of the FST results for the study eye and the fellow eye demonstrate that the responses were similar (see Fig 1). Therefore, this similarity in response indicates that there was no signal in the study. However, contralateral transfer after unilateral intravitreal AAV injection cannot be excluded, as recent studies of similarly administered AAV vectors have demonstrated fellow eye vector transfer and retinal transduction.^11^^,^^12^ Bilateral visual improvements have also been observed after unilateral gene therapy for Leber hereditary optic neuropahthy.^13^

Hereditary retinal diseases are suitable for treatment with gene therapies because of the accessibility of the retina and its immune privilege and compartmentalization, such that gene therapies can be applied with limited immune response or systemic side effects.^5^ Indeed, few patients developed neutralizing antibodies in our study. Moreover, very few systemic adverse effects were reported, and none were believed to be related to AGN-151597 or its administration. Although treatment-related ocular TEAEs were reported, none were serious or severe. Thus our study demonstrated an acceptable safety profile of AGN-151597 in patients with advanced retinitis pigmentosa.

Among the first manifestations of retinitis pigmentosa is difficulty seeing in low-light conditions as rod cells begin to degenerate.^2^, ^3^, ^4^ Full-field stimulus threshold is a measure of whole-field retinal light sensitivity and the lowest luminance stimulus that a patient can see.^8^ Full-field stimulus threshold testing is increasingly being adopted as an endpoint in clinical trials to assess residual vision in patients with severe retinal disease, and its use in clinical settings is growing,^8^ especially since its inclusion as a secondary efficacy measure in the landmark phase III study for the now-approved gene therapy, voretigene neparvovec.^14^ It is especially useful for patients whose vision is so poor as to preclude standard assessment with logarithm of the minimum angle of resolution visual acuity, perimetry, or ERG.^8^^,^^15^

Knowledge of test–retest variability is essential to define a significant change after treatment.^15^ Full-field stimulus threshold test–retest variability of approximately ±3 dB (0.3 log) has been reported,^9^^,^^15^^,^^16^ while a 10 dB (1 log) change in FST is considered clinically meaningful.^14^ The patients who participated in our study had very poor vision ranging from “light perception” to “count fingers,” and the FST assessments (separated by 33–85 days) were consistent, with comparable results obtained between test and retest visits. From these results, we conclude that FST represents a reliable and consistent test that can be effectively used in patients with advanced retinitis pigmentosa and low vision.

The wide heterogeneity of causative mutations in retinitis pigmentosa has hampered efforts to develop genetically targeted treatments.^2^ Most recently, the experimental gene therapy botaretigene sparoparvovec failed to meet its primary endpoint in its phase III LUMEOS trial, joining the list of gene therapy candidates that failed to meet the primary clinical trial endpoint for this disease.^17^^,^^18^

These findings, and ours, are in disappointing contrast with those seen in trials of voretigene neparvovec, an AAV2-based gene therapy that delivers the wild-type version of the *RPE65* gene that encodes an enzyme critical to the retinoid cycle. In a study of voretigene neparvovec in patients with *RPE65*-mediated inherited retinal dystrophy, patients experienced a rapid, 2-log improvement in mean FST to white light by day 30 after a single subretinal administration, and this response was stable for over a year. Other measures of visual function, including the trial’s primary endpoint of bilateral multiluminance mobility test performance, also markedly improved.^14^

Voretigene neparvovec remains the only approved therapy for retinitis pigmentosa. It was approved based on improvement in mobility testing,^14^ whereas our study showed no evidence of improved ambulation in participants treated with AGN-151597. While voretigene neparvovec therapy represents a significant step forward in the treatment of this disabling disease, the proportion of patients who have retinal degeneration associated with pathogenic variants in the *RPE65* gene is very low, and the subretinal administration of voretigene neparvovec is invasive and must be given in specialist hospitals. Availability of an intravitreal therapy that could be given in routine ophthalmic care would be advantageous.

Other optogenetic gene therapies in clinical trials for retinitis pigmentosa include vMCO-1 (sonpiretigene isteparvovec), an intravitreal therapy that has shown meaningful improvements in best-corrected visual acuity versus sham in its ongoing phase IIb/III trial (RESTORE).^19^ Other agents (ZM-02, GS-030, and BS01) are also in ongoing, early-stage clinical trials for retinitis pigmentosa,^20^, ^21^, ^22^ indicating that this remains an active area of research that may offer improved visual function in patients with advanced degeneration, despite the difficulties in bringing such therapies to market.

We acknowledge some limitations to this study. First, the study was single-arm and thus unmasked with no comparator. While this is normal for first-in-human studies, the lack of a comparator means that it is impossible to assess whether the participants’ disease would have worsened further without treatment. However, because AGN-151597 had no meaningful effect on any visual efficacy parameters tested, we are confident in our conclusion that AGN-151597 is not effective for treatment of retinitis pigmentosa. In addition, the number of subjects evaluated in each dose group was too low to identify dose-dependent safety signals from these assessments.

While the precise reasons cannot be determined with certainty, several factors likely contributed to the lack of efficacy. The most probable explanation is insufficient transduction of retinal ganglion cells after intravitreal delivery. The inner limiting membrane is a well-recognized physical barrier to AAV penetration from the vitreous into the retina, and standard intravitreal AAV2 injection requires the vector to traverse this barrier to reach its target cells.^23^ Surgical approaches that bypass or remove the inner limiting membrane have been shown to improve retinal transduction,^24^^,^^25^ but were not used in the study. This may partly explain why subretinal delivery, which bypasses these physical barriers, shows efficacy in subretinally delivered voretigene neparvovec, for example.

Additional contributing factors may include neutralizing antibodies to the AAV2 capsid which were observed in 5 of 14 participants. The presence of AAV2 neutralizing antibodies could have reduced the ability of the vector to transduce retinal ganglion cell had they had the chance to cross the inner limiting membrane. Finally, it is possible that the advanced stage of retinal degeneration in enrolled patients in this study may have limited the number of viable ganglion cells available for transduction.

## Conclusions

In this phase I/IIa trial, a single intravitreal injection of AGN-151597 demonstrated safety in patients with advanced retinitis pigmentosa over the 5-year study period. The absence of safety concerns is encouraging for future development of intravitreal AAV2-based gene therapies in this field. There was no evidence of efficacy of AGN-151597 in slowing the decline in vision in patients with advanced retinitis pigmentosa and low vision, but FST was demonstrated to be a reliable test in these patients.
