# Supplementary material for: Phase I/IIa Study of an Intravitreal Optogenetic Therapy (AGN-151597) in Patients with Advanced Retinitis Pigmentosa
Source: Ophthalmol Sci. 2026 Jul 13;6(9):101323. doi: 10.1016/j.xops.2026.101323 (PMC13475202; doi:10.1016/j.xops.2026.101323)
Supplement: Supplementary file 1 [file mmc1.pdf]

Appendix S1

Full Eligibility Criteria

Inclusion criteria

The following are requirements for entry in the study:

General inclusion criteria

1. Male or female, ≥18 years of age at time of informed consent.
2. Patient has provided written informed consent, and written Authorization for Use and Release of Health and Research Study Information have been obtained prior to any study-related procedures.
3. Patient has the ability to understand and willingness to follow study instructions and is likely to complete all required visits and procedures.
4. Women of childbearing potential must have a negative pregnancy test at Screening and at Baseline, and agree to use an effective form of contraception, or be surgically sterile or postmenopausal (defined as last menstrual period greater than 2 years prior to Baseline). Acceptable methods of contraception include hormonal contraception (i.e., birth control pills, injected hormones, dermal patch or vaginal ring), intrauterine device, barrier methods (diaphragm, condom) with spermicide, or surgical sterilization (tubal ligation).
5. Males must use 2 forms of contraception (including 1 barrier method for 3 months following study treatment administration if their partner is of child-bearing potential) or must be surgically sterile.

Ocular inclusion criteria:

Phase 1 and phase 2a

1. Diagnosis of advanced retinitis pigmentosa defined as:
    a. Clinical diagnosis of advanced retinitis pigmentosa.
    b. Documented retinal electrophysiological evidence of rod–cone photoreceptor degeneration.
    c. Baseline evidence of <10 μV maximal b-wave electroretinography response.
2. Presence of retinal ganglion cells and/or retinal nerve fiber layer on SD-OCT testing.

Phase 1

3. Visual acuity in the study eye of no-better-than hand motion.

Phase 2a

4. If a treatment benefit is identified by the Data Safety Monitoring Committee, visual acuity eligibility criteria will match that of phase 1.
5. If a treatment benefit is **not** identified by the Data Safety Monitoring Committee, visual acuity eligibility criteria will match that of phase 1 for half of the enrolled patients, and visual acuity criteria for the other half of the enrolled patients must range from no-worse-than count fingers to 20/200 vision in the study eye.

Exclusion criteria

Patients will **not** be excluded if the molecular diagnosis underlying their retinitis pigmentosa is not known or if there is presence of macular edema due to advanced retinitis pigmentosa.

However, any one of the following will exclude patients from being enrolled into the study:

General exclusion criteria

1. Participation in any investigational drug or device study within 6 months prior to Day 0.
2. Complicating systemic diseases or clinically significant abnormal baseline laboratory values. Complicating systemic diseases include those in which the disease itself, or the treatment for the disease, can alter ocular function. Examples are malignancies whose treatment could affect central nervous system function (i.e., radiation treatment of the orbit; leukemia with CNS/optic nerve involvement).
3. Immunocompromising diseases, such as hepatitis B, C and HIV, or use of immunosuppressive medications.
4. Females who are pregnant, nursing, planning a pregnancy during the study or who are of childbearing potential not using a reliable method of contraception during their participation in the study.
5. History or current evidence of hypersensitivity to any component of the study treatment, medications, or diagnostic agents used in the study.
6. Immunogenicity assays show presence of neutralizing antibodies to AAV2 above 1:1000 of dilution.

7. Use of antiplatelets and anticoagulants that may alter clotting within 28 days prior to study treatment administration.

8. Any other condition that would not allow the potential patient to complete follow-up examinations during the course of the study and, in the opinion of the investigator, makes the prospective patient unsuitable for the study.

Ocular exclusion criteria

9. History of prior gene or stem cell therapy (ocular or other).

10. Pre-existing eye conditions that would preclude the planned treatment (i.e., injection) or interfere with the interpretation of study endpoints or surgical complications (examples include [but are not limited to] glaucoma, diseases affecting the optic nerve causing significant visual field loss, active uveitis, corneal or lenticular opacities).

11. Cataract surgery, intraocular and/or peri-ocular injection in the study eye within 6 months prior to Day 0.

12. Prior vitrectomy or aphakia in the study eye.
