# Supplementary material for: Phase I/IIa Study of an Intravitreal Optogenetic Therapy (AGN-151597) in Patients with Advanced Retinitis Pigmentosa
Source: Ophthalmol Sci. 2026 Jul 13;6(9):101323. doi: 10.1016/j.xops.2026.101323 (PMC13475202; doi:10.1016/j.xops.2026.101323)
Supplement: Supplementary file 2 [file mmc2.pdf]

Appendix S2

Supplementary Methods for Measuring Safety and Efficacy Parameters

Visual acuity

The visual acuity of each eye was measured using an electronic visual acuity Early Treatment for Diabetic Retinopathy Study (ETDRS) testing protocol, or low vision assessment of count fingers, hand motion, and light perception.

Ambulation

A patient's ability to navigate within a dark room was evaluated. Each participant will be required to walk unaided approximately 10 feet to a wall with a lighted panel. The distance from the canter of the lighted panel to where the patient touched is plotted. If he/she is unable to walk, the test can be conducted with the participant in a wheelchair.

Complete ophthalmic examination

A complete ophthalmic examination was done in both eyes at each study visit. The examination included a slit-lamp biomicroscopic evaluation of the eyelid, eyelid margins and lashes, conjunctiva, cornea, anterior chamber, iris, and lens. The posterior segment and fundus were evaluated through dilated pupils by indirect ophthalmoscopy.

Assessment of intraocular pressure

Intraocular pressure was measured using Goldmann applanation tonometry or a hand-held tonometer (using the same instrument throughout the study for each participant, when possible). If Goldmann applanation tonometry was used to measure intraocular pressure, it must have been done after fundus photography and autofluorescence to prevent fluorescein dye from interfering with the images.

Intraocular pressure was measured in both eyes at every study visit except on the day of AGN-151597 treatment, on which a pre- and post-injection measurement was taken in the study eye only. After the intravitreal injection, the participants remained supine and intraocular pressure was monitored for at least 30 minutes.

Dilated color fundus photography and autofluorescence

A standardized procedure for the collection of single, non-stereo images of the fundus of both eyes was performed.

Spectral domain optical coherence tomography (SD-OCT)

Retinal anatomy of both eyes was evaluated by SD-OCT scans at the Screening visit, Baseline visit, and each scheduled visit starting at Month 1 through Month 24. Additional SD-OCT scans were obtainable at the discretion of the investigator. Each SD-OCT scan was obtained using the study site's preferred imaging system. Use of the same instrument throughout the study for a given participant was recommended.

Object detection and discrimination

Patients performed these 2 tests concurrently, first identifying whether a light displayed on an LED screen could be seen, and then whether a series of standard images (square, circle, triangle, or star) could be identified. Each shape was either red or blue. For each image tested, the shape, color, and threshold intensity was recorded.

Visual evoked potentials

The pattern of visual evoked potentials will be obtained from each eye using a visual evoked potentials stimulator according to standard protocols. The test is performed with the participant sitting in front of an electronic screen, which displays a checkerboard stimulus pattern. The pattern stimulus consists of black and white checks or black and white gratings which abruptly alternate. The electrical responses elicited by the stimulus are recorded and represent the summed response from all active cells.

Electroretinography

Electroretinography testing of both eyes was done using full-field procedures, according to the standard protocol provided by the ERG Standardization Committee of the International Society for Clinical Electrophysiology of Vision (ISCEV) standards. Electroretinography was done at the Screening visit, 6 months, and at 24 months (or early termination visit).

Quality of life (NEI-VFQ-25 scale)

The NEI-VFQ-25 consists of 25 vision-targeted questions that represent 11 vision-related quality of life subscales and 1 additional general health item. The subscales are general vision (Item 2), difficulty with near vision activities (Items 5, 6, 7), difficulty with distance vision activities (Items 8, 9, 14), limitations in social functioning due to vision (Items 11, 13), role limitations due to vision (Items 17, 18), dependency on others due to vision (Items 20, 23, 24), mental health symptoms due to vision (Items 3, 21, 22, 25), driving difficulties (Items 15c, 16, 16a), limitations with peripheral vision (Item 10), limitation with color vision (Item 12), and ocular pain (Items 4, 19). The general health item is Item 1.

The questionnaire was scored using the NEI VFQ-25 scoring algorithm.

Physical and laboratory examinations

A complete physical examination was done at Screening, Month 6, and Month 24 (or the early termination visit), including vital signs (heart rate and systolic and diastolic blood pressure), height, weight, and examination of major organ systems (head/eyes/ears/nose/throat, neck, cardiovascular, pulmonary, abdomen, musculoskeletal/extremities, skin, lymph nodes and neurological). Symptom-specific examinations were done during the study as needed.

Venous samples were collected from a peripheral vein, and laboratory tests were done at each site's preferred certified laboratory. Laboratory testing included the following parameters:

- Complete blood count (CBC): hemoglobin, hematocrit, red blood cell count, mean corpuscular volume, mean corpuscular hemoglobin, mean corpuscular hemoglobin concentration, white blood cell count with differential (neutrophils, lymphocytes, monocytes, eosinophils, basophils), platelet count.
- Chemistry panel: sodium, potassium, blood urea nitrogen (BUN), creatinine, glucose, chloride.
- Liver function tests: albumin, bilirubin, total protein, calcium, alkaline phosphatase, AST (SGOT), ALT (SGPT), triglycerides.
- Serology: human immunodeficiency viral antibody, hepatitis B and C antibody, AAV2 antibody.
- Pregnancy test (for women of childbearing potential): serology beta-human chorionic gonadotropin (b-hCG).
- RST-001 immunogenicity testing.
- Electrochemiluminescence testing.
- Elispot testing.

Standard 12-lead electrocardiograms (ECGs) and posterior/anterior chest x-rays were done and interpreted by a qualified physician.
