# Supplementary material for: Phase I/IIa Study of an Intravitreal Optogenetic Therapy (AGN-151597) in Patients with Advanced Retinitis Pigmentosa
Source: Ophthalmol Sci. 2026 Jul 13;6(9):101323. doi: 10.1016/j.xops.2026.101323 (PMC13475202; doi:10.1016/j.xops.2026.101323)
Supplement: Supplementary file 3 [file mmc3.pdf]

Appendix S3

Ambulation Test Results

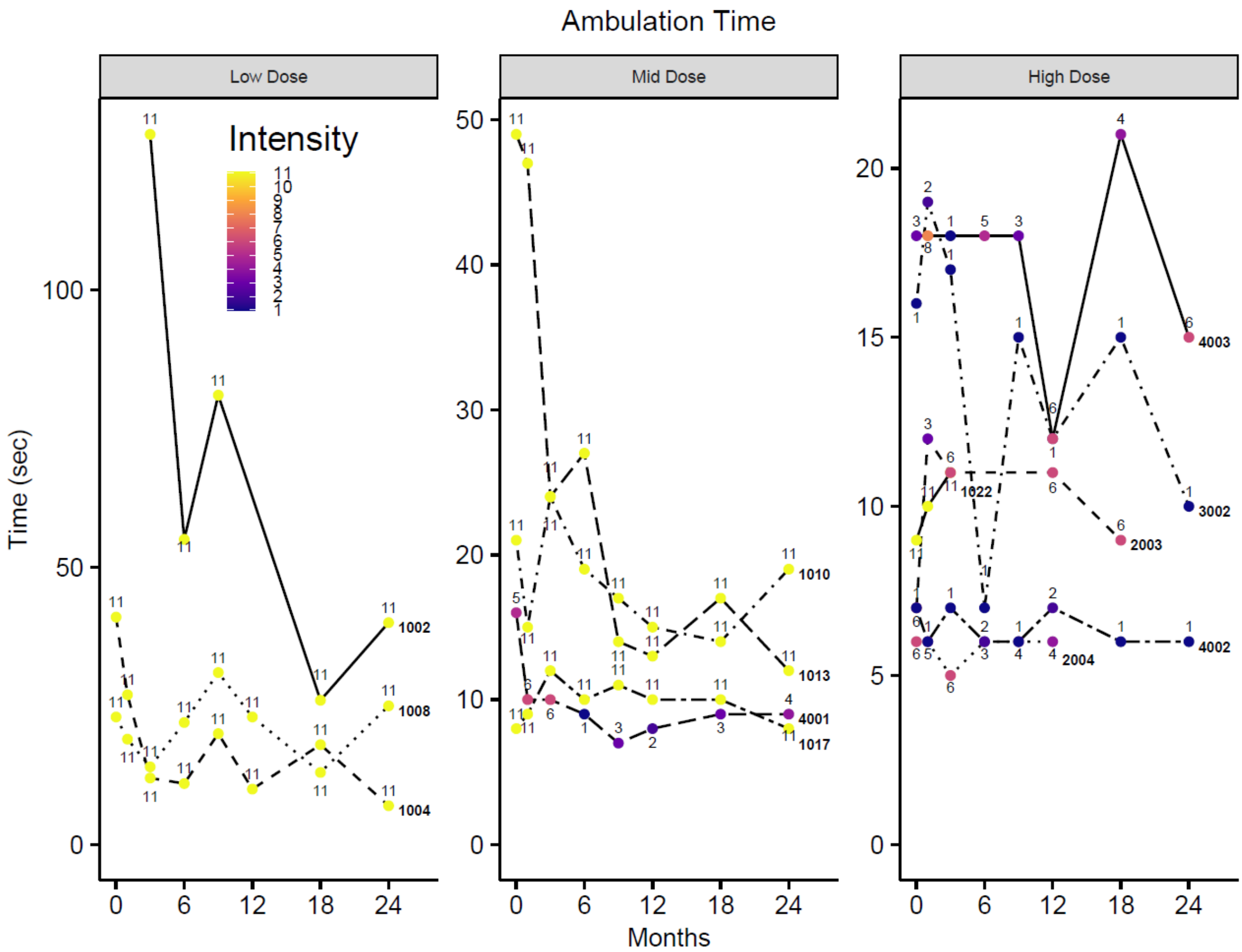

Figure 1. Ambulation test results in participants treated with AGN-151597. Participants were tested 10 times in a dark room. In each trial, the participant (with non-study eye covered) was asked to walk 10 feet toward a lit panel and touch the center of the panel. The intensity of light was set at the lowest luminance visible to the participant. The time from the start to when the participant stopped walking was recorded. Results for individual participants are plotted and identified by participant number. There was no clear change in participants' ambulation time during testing over the course of the study.

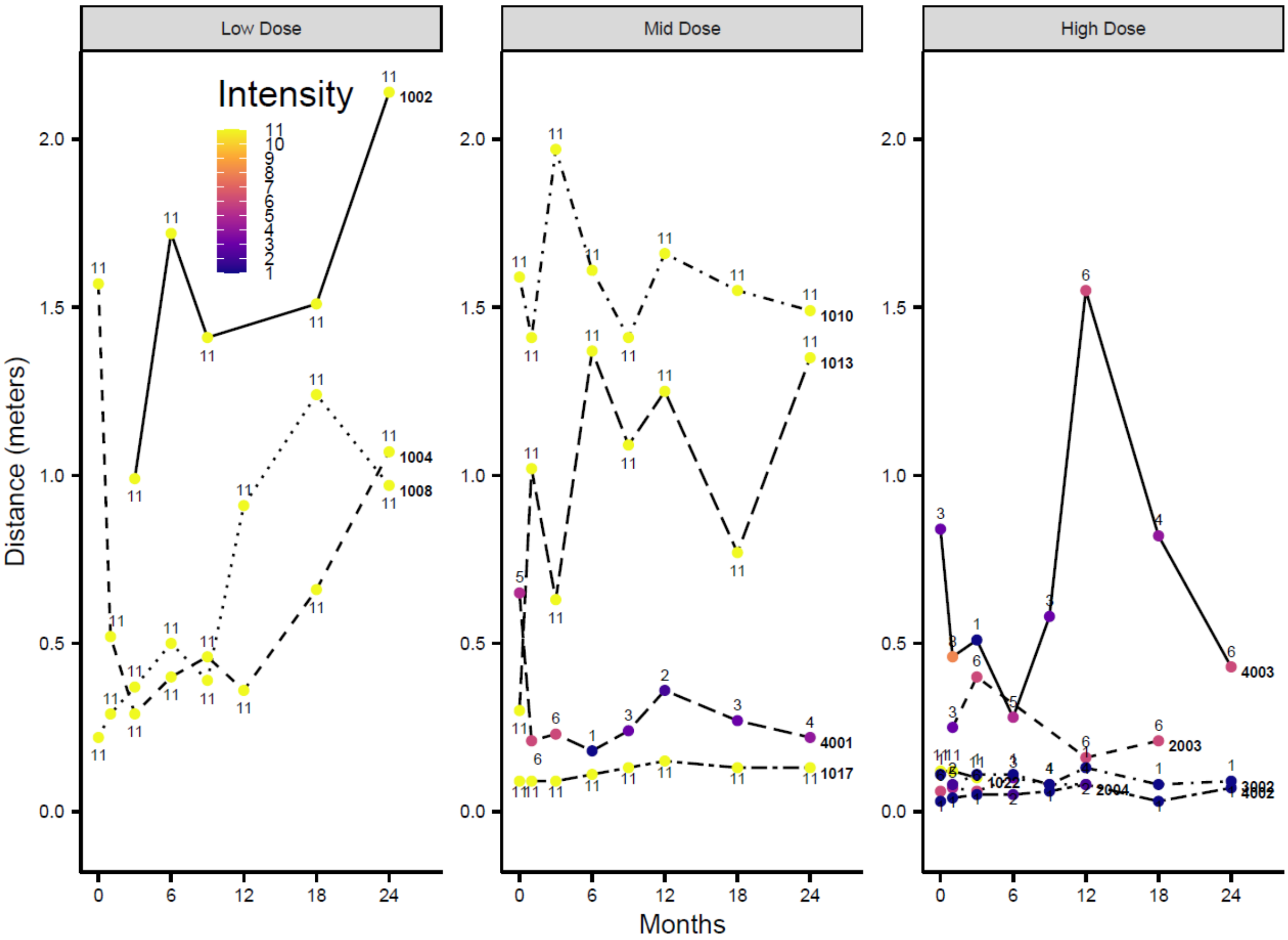

Figure 2. Ambulation test results in participants treated with AGN-151597. Participants were tested 10 times in a dark room. In each trial, the participant (with non-study eye covered) was asked to walk 10 feet toward a lit panel and touch the center of the panel. The intensity of light was set at the lowest luminance visible to the participant. Results for individual participants are plotted and identified by participant number. There was no clear change in participants' ambulation distance during testing over the course of the study.
